# Smartphone Addiction Inventory (SPAI): Translation, adaptation and validation of the tool in Spanish adult population

**DOI:** 10.1371/journal.pone.0205389

**Published:** 2018-10-17

**Authors:** Conchín Simó-Sanz, M.ª Luisa Ballestar-Tarín, Antonio Martínez-Sabater

**Affiliations:** 1 Hospital General Universitario de Valencia, Valencia, Spain; 2 Nursing Department, University of Valencia, Valencia, Spain; North Carolina Neuropsychiatry Clinics, UNITED STATES

## Abstract

The wide functionality and the vast range of attributes offered by smartphones has led to a substantial increase in the average amount of time these devices are used per day. An excessive use of these tools has been shown to result in symptomatology similar to psychological disorders caused by substance addiction. In Spain, smartphone use has risen exponentially but the effects of this increase remain unclear. Therefore, an instrument is required to help determine the extent of smartphone addiction in the Spanish population. The Smartphone Addiction Inventory (SPAI) is a valid and reliable mean to identify and measure smartphone addiction and so, the aim of this research is the translation and adaptation of SPAI to Spanish, as well as the analysis of its psychometric properties in a Spanish adult population of 2,958 adults, at the University of Valencia. A multiphase-interactive model has been used, based on classical translation–back-translation methods to translate and adapt the SPAI. Moreover, a confirmatory factor analysis to verify that the inventory showed acceptable goodness of fit indices (χ^2^_293_ = 4795.909, Comparative Fit Index = 0.927, Tucker–Lewis Index = 0.919, Root Mean Square Error of approximation = 0.072, and Standardised Root Mean square Residual = 0.051) has been carried out. Also good reliability has been found for the global inventory (Cronbach’s alpha = 0.949), and each of its corresponding factors: compulsive behaviour, functional impairment, abstinence, and tolerance (Cronbach’s alpha = 0.856, 0.888, 0.855, and 0.712, respectively). Hence, the SPAI has been adequately translated and adapted for its use in Spain and therefore it is a useful tool for evaluating the degree of smartphone addiction in the Spanish adult population.

## Introduction

According to the International Telecommunication Union, Spain is placed 26th in the Information and Communication Technologies Development Index ranking [1]. This worldwide valid index is in charge of controlling and comparing Information and Communication Technologies (ICT) evolution among countries. Smartphone use in Spain has been spread to all areas of work, social, and family life. The presence of these devices in Spanish households has increased by 9.5% over the last ten years [2], and it is estimated that an average user consults their smartphone about 150 times a day and that 80% of users sleep next to it [3]. The high presence of this device has motivated an increasing number of researches to investigate the potential dangers of its use [4], due to some negative repercussions for health that have already been shown following their intense daily use [5–7].

The abuse of smartphones gives rise to symptoms closely resembling those seen in patients with substance addictions. These include lack of impulse control [8–10], tolerance [11,12], dependence [13], anxiety related to service connectivity [14], changing states of mind [15], and interferences in daily life [16,17], sleep [18], and personal relationships [9,19]. The similarities between these symptoms and those of other addictive disorders have created controversy as to whether to treat smartphone abuse as a mere situational problem or if some cases should be treated as a true addictive disorder [20–23]. The main mental disorder and disease classification manuals do not currently consider ‘smartphone addiction’ [24,25] and its possible future inclusion would first require more investigation.

Billieux [9] considers “problematic mobile phone use” (PMPU) as an inability to regulate one’s use of the mobile phone, which eventually involves negative consequences in daily life. Researches developed in Spain by Jenaro et al. [10] and López-Fernández [26] found values of PMPU that oscillate between 7.99% and 12.5% among people aged over the age of 18. In these studies, authors have used scales elaborated by themselves such as COS (Cell-Overuse Scale) and the SAS-SV (Smartphone Addiction Scale-Short Version) translation and adaptation. This last one was created by López-Fernández [26], starting from the Smartphone Addiction Scale of Kwon, Kim, Cho, & Yang [27].

Smartphone use has also substantially increased at global level [28] and as a result, different types of scales for measuring it are becoming available in the literature [29] and are being cross-culturally adapted for its use in different populations. These include Leung’s Chinese Mobile Phone Addiction Scale [30] derived from Bianchi and Phillips’ Problem Mobile Phone Use Scale [8] and the recent French and Spanish versions of the Smartphone Addiction Scale-Short Version by López-Fernandez [26] itself adapted from the Smartphone Addiction Scale by Kwon et al. [27]. Such cross-cultural adaptation and validation not only allows instruments to be produced with psychometric qualities that are comparable with the originals and facilitate international comparison of results, but also helps to avoid the irrational creation of new scales with similar purposes [31] and the formation of connections between different lines of research on addictions to technology.

The choice of SPAI to carry out this study [29] is mainly due to its factor analysis (Compulsive behaviour, Functional impairment, Withdrawal and Tolerance). The four dimensions of the SPAI constitute some of the DSM-V substance abuse diagnosis criteria. This circumstance makes SPAI especially interesting for the establishment of a parallelism between the addictive use of the smartphone and the addictive disorder by substances [32].

In this study we translated the Smartphone Addiction Inventory (SPAI) from Lin et al. into Spanish, and we also cross-culturally adapted and validated it [33]. Our hypothesis is that the Spanish version of SPAI will be a valid and a reliable instrument to measure the addictive use of the smartphone in Spanish adult population.

## Materials and methods

### Study design and ethical aspects

We translated the SPAI into Spanish and performed cross-cultural adaptation and validation of the resulting instrument following a multiphasic model similar to that used in the adaptation of other instruments, such as “International Classification of Impairments, Disabilities and Handicaps” (ICIDH), promoted by the World Health Organization [34]. Furthermore, the University of Valencia postgraduate studies ethics committee reviewed and approved this study, and all the participants gave their written consent to their voluntary and anonymous involvement prior to completing the inventory.

### The Smartphone Addiction Inventory

The SPAI is a self-administered inventory created by Lin et al. [33] which aims to identify the characteristics of addiction in the context of smartphone use. It includes 26 items scored using a 4-point Likert scale, where 1 represents strongly disagree, 2 represents slightly disagree, 3 represents somewhat agree, and 4 represents strongly agree. Confirmatory factor analysis (CFA) identify four dimensions: compulsive behaviour, functional impairment, abstinence, and tolerance. Internal consistency was high: the overall Cronbach’s alpha score was 0.94 for the global scale and 0.87, 0.88, 0.81, and 0.72 for each of its respective factors.

### Translation and cultural adaptation to Spanish

We used a multiphase-interactive model, based on classical translation–back-translation methods [35]. Four independent translators collaborated (T1, T2, RT1, RT2). First, the English SPAI was translated into Spanish (by two translators, T1 and T2).

Then a monolingual Spanish discussion group was created with a convenience sample of four participants. A member or the research team gave them the translation prepared by T1 and T2 and this person conducted a personal and structured survey in order to assess the understandability of all the items and the adequacy of the terms and expressions to our cultural environment.

At the same time, a group of three bilingual experts were asked to agree upon the final Spanish version. The following numerical code was used to assess T1 and T2 translations: ‘0’ meant that the translations by T1 and T2 were similar and no doubts were raised about the vocabulary or the content; ‘1’ meant that T1 and T2 differed in some words and the team of bilingual experts identified slight discrepancies in translation; ‘2’ meant that with these great discrepancies it was impossible to obtain an equivalent translation to Spanish language.

The group of bilingual experts reviewed the translation of the coded items with value “1” and prepared a consensual translation, helped with the contributions of the monolingual group.

The Spanish version developed after the bilingual experts’ consensus was then translated back into English by two different translators (RT-1 AND RT-2) and the resulting text was assessed for any expression that no longer adequately conveyed the original meaning of the instrument.

### Sample

The new Spanish version of the SPAI was completed by 2,958 participants from the University of Valencia (Spain), including students, professors and administrative staff. Participants were limited to those aged at least 18 years and those who owned a smartphone. An electronic web-based version was disseminated by e-mail on April 5, 2017 and data were collected during 23 days; the system required completion of all the items and did not allow duplicate answers.

### Data analysis

CFA is a statistical method used to validate scales because it stablishes the number of factors underlying the measures, the items corresponding to each factor, and the relationships existing among them. In this study we used the statistical program R v.3.4.1 [36] to carry out the CFA. We considered that due to the large sample size and high number of dependent variables (26 items), it was most suitable to apply the ‘weighted least squares with mean and variance’ (WLSMV) adjustment. Then, the regression coefficients and goodness-of-fit test results resulting from the chi-square (χ^2^) test were analysed: Comparative Fit Index (CFI), Tucker–Lewis Index (TLI), Root Mean Square Error of Approximation (RMSEA), and Root Mean-Square Residual (RMSR). Overall model fit was judged using the following cutoff values: for the CFI and TLI, values larger than 0.95 are considered as indicators of good fit [37–39] and values between 0.90 and 0.95 are usually interpreted as indicators for an acceptable fit. For the RMSEA, values lower than 0.05 indicate good fit, values between 0.05 and 0.08 indicate acceptable model fit, and values greater than 0.10 suggest a poor model fit [40]. For RMSR, values lower than 0.08 indicate good fit [40]. In this study, we also measured the internal consistency of the inventory using the Cronbach’s alpha coefficient.

## Results

### Sample characteristics

The study sample comprised 2,958 people, 65.3% female. The average age was 27.96 ± 12.1 years (26.43 ± 10.52 for female group and 30.8 ± 14.2 for male group). We also studied selected sociodemographic data: I) the highest qualification level; II) smartphone use patterns; III) time of smartphone use; and IV) monthly economic cost of smartphone use; these results are shown in Table 1.

**Table 1:** Selected sociodemographic characteristics of the study sample.

Characteristics of the sample	N	(%)	Characteristics of the sample	N	(%)
Gender			Money spent on smartphone per month		
Male	1025	34.6	Less than 20 euros	1737	58.8
Female	1933	65.3	More than 20 euros	1221	41.2
Highest qualification level			Smartphone usage time per day		
Primary education	10	0.3	Less than 1 hour	240	8.1
Secondary education or vocational training	515	17.4	Between 1 to 4 hours	1822	61.6
University bachelor’s or postgraduate degree	2433	82.2	More than 4 hours	896	30.3

The average score of the SPAI Spanish version obtained by the sample was 46.96 ± SD 13.6. Women score higher than men: 40.42±10.12 vs. 39.21±10.43, being these differences statistically significant when analyzed with Mann-Whitney test (p<0.05). Table 2 shows the average scores according to age group, that have also shown to be statistically significant (p<0.001, Kruskall Wallis test)

**Table 2:** Average score of the SPAI Spanish version according to age.

Age groups	SPAI Score	
	Mean	SD
18–25 years	49	11.60
26–35 years	45.57	11.31
36–45 years	43.77	11.01
>46 years	39.8	11.51

### Translation and cross-cultural adaptation

From the 26 items on the scale, 12 of them were classified with code "0" since both translated versions were very similar and maintained the conceptual equivalence with the original version. For example, in items 8, 9, 14 and 21, both translations differed only by the position of the words within the sentence and so were considered as similar. However, 14 items (3, 4, 5, 10, 11, 12, 13, 15, 17, 18, 19, 20, 25, and 26) were classified with code ‘1’; this was either because there were small differences in some of the terms used. For example, T1 translated item 5 (in Spanish) as something like “I feel energetic about using my smartphone regardless of how tired I am” while T2 translated the same item (in Spanish) as something similar to “I feel good using my cell phone, although I have sometimes felt tired”. There were no items classified as ‘2’.

### Confirmatory factor analysis

**Fig 1:**
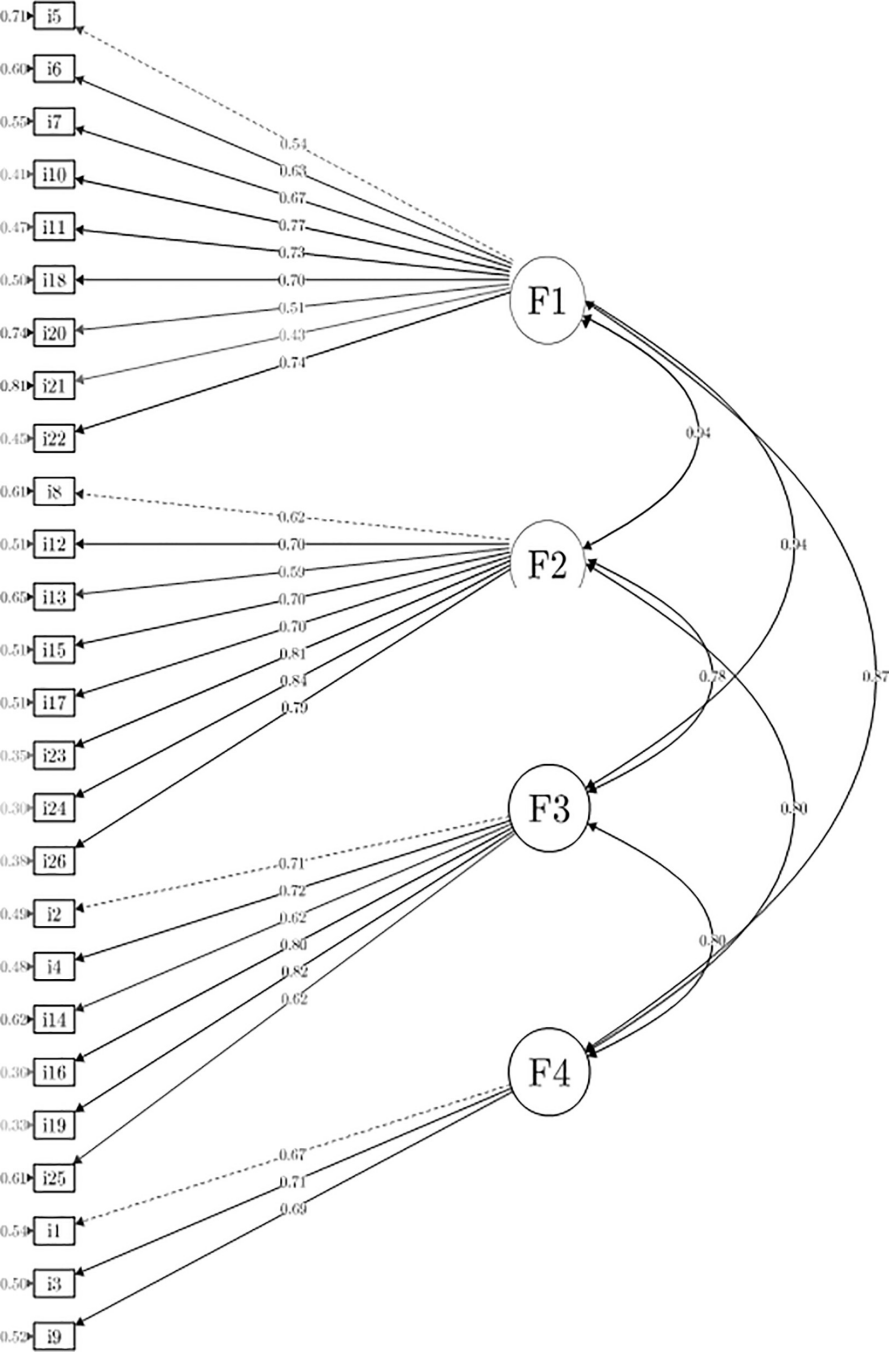
Graphical representation of our confirmatory model.

The value of 0.96 of Kaiser–Meyer–Olkin test of sampling adequacy and a significance of Bartlett´s test of sphericity (p<0.001) indicated the suitability of CFA. Table 3 shows the factor loadings results for each item. The goodness-of-fit indexes for the confirmatory model were as follows: (χ^2^_293_ = 4795.909; *p* < 0.000; CFI = 0.927; TLI = 0.919; RMSEA = 0.072). The Cronbach’s alpha coefficient was 0.949 for the global inventory and 0.856, 0.888, 0.855, and 0.712 for each of the corresponding factors (compulsive behaviour, functional impairment, abstinence, and tolerance, respectively) indicating that the results were very reliable. Fig 1 shows a graphical representation of the confirmed model based on our data.

**Table 3:** Factor loadings of Spanish version of the Smartphone Addiction Inventory according to the model presented by Lin et al. [33].

Item			Standard error	Compulsive Behaviour	Functional Deterioration	Abstinence	Tolerance
**5**	I feel very vigorous when I use my smartphone, regardless of my level of fatigue.		-	0.542				
**6**	I use smartphone for longer and spend more money on it than I intend to.		0.038	0.631			
**7**	Although using my smartphone has had negative effects on my interpersonal relationships, the amount of time I spend on the internet remains unreduced.		0.037	0.674			
**10**	I feel distressed or down once I stop using my smartphone for a certain period.		0.039	0.766			
**11**	I cannot control the impulse to use my smartphone.		0.036	0.731			
**18**	My recreational activities are reduced because of my smartphone use.			0.705			
**20**	My life would be joyless if I did not have a smartphone.		0.035	0.508			
**21**	Using my smartphone has placed me in dangerous situations: for example, I have used it while crossing the road or while driving.		0.034	0.431			
**22**	I try to spend less time on my smartphone, but my efforts are in vain.		0.038	0.740			
**8**	I have slept less than 4 h more than once because of my smartphone use.		-		0.621		
**12**	I find myself using my smartphone at the cost of socialising with my friends.		0.034		0.700		
**13**	I get aches and soreness in my back or eye discomfort caused by excessive smartphone use.		0.029		0.591		
**15**	My smartphone use has had certain negative effects on my schoolwork or job performance.		0.033		0.704		
**17**	My interaction with family members is decreased because of my smartphone use.		0.034		0.701		
**23**	My smartphone use is a habit and as a result my sleep quality and overall sleep time has decreased.		0.034		0.806		
**24**	I need to spend an increasing amount of time on my smartphone to achieve the same satisfaction as before.		0.036		0.838		
**26**	I feel tired during the daytime due to late-night use of my smartphone.		0.032		0.789		
**2**	I feel uneasy once I stop using my smartphone for a certain period.		-			0.712	
**4**	I feel restless and irritable when my smartphone is unavailable.		0.019			0.724	
**14**	The first thing I think about when I wake up each morning is using my smartphone.		0.019			0.616	
**16**	I feel like I am missing something when I stop using my smartphone for a certain period.		0.020			0.801	
**19**	I feel the urge to use my smartphone again immediately after I stop using it.		0.029			0.818	
**25**	I cannot sit down to eat without having my smartphone with me.		0.024			0.625	
**1**	I have been told more than once that I spend too much time using my smartphone.		-				0.675
**3**	I find that I am hooked on my smartphone increasingly longer periods.		0.029				0.711
**9**	Over the past three months I have substantially increased the amount of time I spend using my smartphone.		0.029				0.694

### Validity analysis

The questionnaire included a question about self-perception of Smartphone addiction, which the participant evaluated from 1 to 10. The relationship between this answer to the question and the global scores obtained in the SPAI for construct validity analysis. A direct lineal correlation between both measurements was found and it was statistically significant (rho = 0.595, p<0.001).

We also employed SPAI results according to participants’ age and gender for the criterion validity analysis; finding that both young people and women achieved higher results on the SPAI scale, as we could expect after analyzing the literature.

## Discussion

In this study we have obtained the Spanish version of the “Smartphone addiction Inventory” following all the phases of the translation and adaptation process and it has confirmed with appropriate indexes of validity and reliability the model of four factors (compulsive behaviour, functional impairment, withdrawal and tolerance) raised by Lin et al. [33] in their study.

In the reliability analysis, the “Tolerance” factor obtained the lowest value of Cronbach’s alpha (0.766). Despite being the lowest one, it is a value that indicates acceptable internal consistency of the factor, and also improves the result found in the version of Lin et al. [33] in which it presents a Cronbach’s alpha of 0.712.

Tolerance is a diagnostic criterion of any addictive behaviour to substances, although this dimension is questioned in regard to the use of the Smartphone [41], since its use is sometimes not voluntary but a consequence of the obligatory nature of specific circumstances such as work or family.

One of the most important characteristics of SPAI is its four factors that emerge after the CFA, since they allow establishing similarities between addictions to substance and behavioral addictions.

This comparison is established not so much because of the harmful consequences for health that mobile addiction may have, but because of the fact that in situations of excessive use, symptoms related to abstinence have come to appear, such as the compulsive use and the desire and constant thinking.

This last question is important to follow the line of the inclusion of behavioral addictions within the main classifications of mental disorders (DSM-V AND ICD-10). The Spanish version of SPAI has proven to be a capable tool of discerning addictive use through at least 3 reliable factors involved in any addictive behavior: compulsive behavior, functional impairment and abstinence.

Most questionnaires on mobile phone use that are currently available are now obsolete because of the speed of evolution of this type of device. For example, it would now be useless to evaluate SMS use [15] because of its virtual replacement with instant messaging. Likewise, evaluating only the voice-call functionality of smartphones without taking their intrinsic connection to the Internet use into account would now be inappropriate.

Prior to conducting this study, only two scales were available in Spanish language for the adult population: The *Cell-Phone Over-Use Scale* (COS) by Jenaro et al. [10] and The Questionnaire of Experiences Related to the Use of Mobile (CERM) of Beranuy et al. (2009)[42]. However, none of them seeks to assess the addiction or the disorders that this term implies (Tolerance, dependence, abstinence, craving, etc.) The SPAI-SPAIN version has shown to be an appropriate tool to screen smartphone addiction because it is current, simple and fast based on the device’s characteristics (portability, multifunctionality and connectivity). In addition, it has proven to be valid and reliable in its previous adaptations to other countries (Taiwan, Italy and Brazil) which allows us to expand the population comparisons.

The new possibilities offered by smartphones are evaluated with more current instruments, however it is important that these could be applied in populations with a wider age range because most recent studies have focused only on teenagers [43–45]. Nevertheless, Lu et al. [46] suggested that the psychological dependence created by some smartphone functionalities is not exclusive to teenagers, but also affects adults. Thus, many studies are now starting to include higher age ranges [16,17, 47] in order to fully assess the enormous addictive potential of smartphones, as we have developed in this research.

The sample we used in this current study was large and the age was 27.96 ± 12.1 years, higher than in previous studies and in all the validation studies of SPAI carried out in different countries. If we compare the average score obtained from SPAI-SPAIN (46.96 ± SD 13.6) with SPAI (original version) (51.31 ± SD 11.77), we can find that Spaniards achieve a lower average score. If we analyze our results by age group, we can affirm that Spanish youngers get a higher SPAI score than in the original one, which coincides with the results of other studies [48]. All things considered, all age groups get high scores, reflecting the impact of this device on our daily lives. These results show the need to develop studies in any age group, especially in the older ones [49].

The SPAI has already been validated in Italian [50] and Brazilian [51] -the SPAI-I and SPAI-BR, respectively-. The authors of the SPAI-I confirmed the original model, but goodness-of-fit indices were ambiguous and so, they chose a five-factor model (comprising *time spent*, *compulsivity*, *daily life interference*, *craving*, and *sleep interference*) to obtain the following fit indices (χ^2^_68_ = 118.834; *p* < 0.000; CFI = 0.94; TLI = 0.97; RMSEA = 0.05; and SRMR = 0.87). The SPAI-BR originally contained four factors (*compulsive behaviour*, *functional impairment*, *withdrawal*, *tolerance*) with acceptable fit indices (χ^2^_166_ = 626.482, CFI = 0.9938, TLI = 0.931, RMSE = 0.052). However, the authors opted for a unifactorial model because the internal consistency of the factors was low, and in the case of the tolerance factor, the Cronbach’s alpha value was less than 0.7. Finally, they defined a unifactorial structure, according to Goodman’s criteria of behavioural addictions, used as a basis for the construction of addictive disorders’ diagnostic criteria of ICD-10 and DSM-5, to obtain the following fit indices: χ^2^_299_ = 767.861; CFI = 0.913; TLI = 0.905; RMSE = 0.061; and WRMW = 1.465. Similarly, in our Spanish version of the instrument (SPAI-Spain), the tolerance factor was not internally consistent (the Cronbach’s alpha value was 0.74) so we agree with Lin et al. [33] that the reliability of this factor in these types of addictions should be called into question.

Our translation and validation of the SPAI fulfilled all the requirements of the translation and transcultural adaptation process [35] and so the SPAI-Spain can now be used to measure the degree of smartphone addiction in the general Spanish population, including adults. Its validity and reliability indices were good and thus, it can discern addictive use by means of at least three reliable factors known to be involved in all addictive behaviour: compulsive behaviour, functional deterioration and abstinence.

A limitation of our study is determined by the selection of the studied population since, although the sample size finally obtained is high, a stratified selection has not been made, so that the participants do not constitute a representative sample of the Spanish population.

Another limitation is that we have not identified the smartphone’s models used by the surveyed because these devices are in continuous development. It is very difficult because it would be necessary to develop specific questionnaires for each type of device, which is unaffordable.

In conclusion, the SPAI-Spain can be used to assess the degree of smartphone addiction within a preventive program or to evaluate the evolution within a treatment process, regardless of the treatment type (e.g., cognitive–behavioural, pharmacological, etc.). Its use in clinical practice should also reveal information about which of the four factors identified in this study and related to the addictive use of smartphones is most affected at the individual level. Thus, this type of research can help to increase our knowledge of technology addictions within a framework of possible addictive disorders.

## Supporting information

S1 File Data used in study. (SAV)
